# The opportunity for e-mental health to overcome stigma and discrimination

**DOI:** 10.1192/j.eurpsy.2024.1141

**Published:** 2024-08-27

**Authors:** K. Subramaniam, A. Greenshaw, A. Thapliyal

**Affiliations:** ^1^Global Medical Affairs, Viatris, Auckland, New Zealand; ^2^Dept of Psychiatry, University of Alberta, Edmonton, Canada; ^3^e-Mental Health International Collaborative, Auckland, New Zealand

## Abstract

**Introduction:**

Many with mental illness do not seek treatment, often due to stigma; be it public, self, or institutional type. To improve outcomes, stigma needs addressing.

**Objectives:**

Understand the opportunity for e-mental health to help overcome stigma and, to provide an expert opinion to foster its adoption.

**Methods:**

We conducted literature searches using the terms ((mental health) AND ((stigma) OR (discrimination))) AND (((((digital tools) OR (digital services)) OR (healthcare apps)) OR (digital solutions)) OR (digital technology)), limited to 2007 – 2023, identifying 223 citations, 9 of which were relevant for this evaluation, including 4 systematic reviews (** Table 1**).

**Results:**

Literature reports suggest that e-mental health may be useful for addressing stigma and reducing the treatment gap. While it was not consistently as good as face-to-face services, e-mental health tools were frequently shown to be effective in reducing stigma, improving mental health literacy, and increasing help-seeking behaviors. Tools included web-based breathing, meditation, and CBT; suicide prevention apps; and online videos and games. Experts from a 2022 global Think Tank session convened by eMHIC, opined and emphasised that embracing e-mental health must not leave people behind nor reinforce inequality and that structural barriers must first be acknowledged and overcome. Creating a shared understanding of the challenge and of terminology is essential, as is codesigning any solution together with people with lived experience.

**Table 1.** Systematic literature reviews

Study	Interventions	Findings
SLR + meta-analysis, 9 studies, n=1916 (Goh et al. Int J Ment Health Nu 2021;30:1040–1056)	Web-based program MIDonline AboutFace BluePages MoodGYM MHFA eLearning Beyond Silence	Online vs offline: similarly effective for reducing public stigma
SLR, healthcare setting (Pospos,et al. Acad Psychiatry 2018;42:109–120)	Breath2Relax Headspace Meditation Audios MoodGYM Stress Gym Virtual Hope Box Stay Alive	Identified tools provide a starting point to mitigate burnout, depression, and suicidality
SLR, 13 interventions for stigma (Johnson, et al. Indian J Psychol Med 2021;44:332–340)	Web-based, psychoeducation interventions Online games Mobile app	Most interventions increased help-seeking
SLR + meta-analysis, 9 RCTs, n=1832 (Rodriguez-Rivas, et al. JMIR Serious Games. 2022; 10:e35099)	Video games Virtual reality Videoconferencing and online chat	Interventions had a consistent effect on reducing public stigma

**Conclusions:**

Published data suggest that e-mental health is promising to reduce stigma and discrimination, with the potential to foster help-seeking and treatment engagement. Adoption requires attention to derailers and must foster inclusivity. There is an imperative to adopt e-mental health, especially evidence-based solutions.

**Disclosure of Interest:**

K. Subramaniam Employee of: Employee of Viatris, A. Greenshaw: None Declared, A. Thapliyal: None Declared

